# The Clinicopathological Correlation and Outcome of Glomerulonephritis With Crescent: A Single-Center Study

**DOI:** 10.7759/cureus.54996

**Published:** 2024-02-26

**Authors:** Rajeshwar Rao, Prit P Singh, Om Kumar, Amresh Krishna, Prem S Patel

**Affiliations:** 1 Nephrology, Indira Gandhi Institute of Medical Sciences (IGIMS), Patna, IND

**Keywords:** glomerulonephritis with crescent, remission, treatment response, renal biopsy, crescent age

## Abstract

Background: There is a lack of standardized treatments for patients with less than 50% crescents observed in their renal biopsies. This study aimed to analyze the crescent percentage, clinicopathological characteristics, and renal prognosis of glomerulonephritis (GN) cases with at least one crescentic lesion.

Materials and methods: This retrospective cohort study was conducted at the Indira Gandhi Institute of Medical Sciences, Patna, from January 2016 to December 2020. Consecutive patients (aged between 18 and 65 years) with renal biopsy findings suggestive of GN and at least one crescent were included in the study. Demographic details and clinical presentation were collected from the medical records.

Results: A total of 145 patients were included. The mean (standard deviation (SD)) age was 33.06 (11.739) years. Hemoptysis was significantly higher in the ≥50% crescent group (P=0.011). Rapidly progressive glomerulonephritis (RPGN) was significantly higher in the ≥50% crescent group (P<0.001). There was a significant difference observed in mean creatinine (P=0.001), mean crescents (P<0.001), and mean urine polymerase chain reaction (PCR) (P=0.031). Antineutrophil cytoplasmic antibody (ANCA)-associated vasculitis was significantly higher in the ≥50% crescent group (P<0.001).

Complete remission decreased as crescents increased. In GN with crescent, the presence of fibrous crescents (≥50%) is associated with a higher rate of treatment resistance (100%) compared to fibrocellular (58.33%) and cellular crescents (6.25%). In the ≥50% crescent group, death was significantly higher in patients with fibrous crescent age (57.14%).

Conclusion: Crescent percentage and crescent age were found to be significantly related to greater risk of renal failure and resistance to treatment.

## Introduction

Rapidly progressive glomerulonephritis (RPGN) is identified by a more than 50% decline in glomerular filtration rate (GFR) within three months, in kidney function, typically stemming from various conditions that lead to the development of crescent-shaped structures visible in renal biopsies. Consequently, it is also referred to as glomerulonephritis (GN) with crescent in pathological terminology [1,2]. RPGN is a very rare condition worldwide [3]. The incidence rate in the United States stands at about seven cases per one million persons per year, while the United Kingdom reports two cases per one million [4]. It necessitates early intervention to prevent progressive renal failure or death [1]. GN with crescent is one of the most common causes of morbidity and mortality due to irreversible kidney failure [5]. It occurs in a wide variety of systemic and primary glomerular diseases secondary to immunological or non-immunological etiologies [6].

Renal biopsy serves as the gold standard diagnostic procedure for GN and offers valuable predictive insights for patients exhibiting crescent formation in their biopsy specimens. The disease's severity correlates with the proportion of crescent formation evident in the renal biopsy [3]. An extent of crescent formation exceeding 80% indicates an advanced stage of renal failure and a limited response to treatment. Conversely, crescent formation below 50% is associated with improved kidney survival and treatment responsiveness [7-10]. GN with crescent can be categorized into three distinct disease groups determined by the immunofluorescence microscopic pattern identified in kidney biopsy: linear, granular, and pauci-immune. A linear pattern indicates the presence of anti-glomerular basement membrane disease, while granular staining is characteristic of immune complex-mediated conditions, such as lupus nephritis and post-infectious GN [1]. Pauci-immune GN (pauciGN) is a constituent of systemic small vessel vasculitis, such as granulomatosis with polyangiitis [11]. In the adult population, pauci-immune GN (pauciGN) is the prevailing condition, especially among white individuals, males, and those aged over 65 years [8,12]. Conversely, GN with crescent in children more frequently exhibits an immune complex-mediated etiology [13].

The etiology influences the clinical manifestations and immunopathological features of GN with crescent. Hence, determining the etiology is crucial for initiating the proper treatment [14]. Untreated RPGN promptly results in an accelerated deterioration of renal function occurring within weeks to months, underscoring the critical need to initiate treatment promptly [4]. Therapeutic intervention in GN with crescent is therefore required to prevent irreversible alterations in glomerular structure.

There is a notable heterogeneity in the etiology and outcome of GN with crescent [15]. There are well-known treatment protocols for diffuse GN with crescent, but standard treatment for patients who have fewer crescents in their renal biopsies is limited [3]. The present study aimed to analyze the crescent percentage, clinicopathological features, and renal prognosis of GN cases that exhibited at least one crescentic lesion.

## Materials and methods

Study design

This retrospective cohort study was conducted at the Department of Nephrology, Indira Gandhi Institute of Medical Sciences, Patna, between January 2016 and December 2020. The report of patients who underwent renal biopsy was reviewed.

Ethical approval

This study was conducted in accordance with ethical principles that are consistent with the Declaration of Helsinki. The study protocol was approved by the Institutional Review Board/Ethics Committee (282/IEC/IGIMS/2021). Written informed consent was obtained from all the patients prior to study commencement.

Inclusion and exclusion criteria

Consecutive patients (aged between 18 and 65 years) who exhibited renal biopsy features suggestive of GN with at least one crescent were enrolled in the study. Known cases of chronic kidney disease and pregnant females were excluded from the study.

Data collection

Patients were divided into three groups based on the percentage of crescents, group I (≤25% crescents), group II (26%-49% crescents), and group III (≥50% crescents), which was further subdivided based on crescent age into cellular, fibrocellular, fibrous, and cellular with fibrocellular crescentic group. The demographic data and clinical presentation (nephritic range proteinuria, sub-nephrotic proteinuria, associated hypertension, etc.) were collected from the medical records. All the patients were followed up for a period of a minimum of 12 months for the evaluation of response to treatment and evaluation of renal outcome. All patients were treated according to the Kidney Disease: Improving Global Outcomes (KDIGO) practice guidelines for GN [16] or standard prevailing guidelines.

Statistical analysis

Data were analyzed using the Statistical Package for the Social Sciences (SPSS) version 25.0 (IBM SPSS Statistics, Armonk, NY). Qualitative data were presented as numbers and percentages, while quantitative data were presented as mean (standard deviation (SD)). The normal distribution of quantitative data was assessed using the Shapiro-Wilk test. A comparison of qualitative and quantitative variables between the groups was done using the Mann-Whitney U test and chi-square test, respectively. A P value of <0.05 was considered statistically significant.

## Results

A total of 145 patients were included, of which 67 patients had ≤25% crescents, 35 patients had 26%-49% crescents, and 43 patients had ≥50% crescents. The mean (SD) age of the patients was 33.06 (11.739) years. The majority of patients were female (57.24%). There was no significant difference observed in age (P=0.540), gender (P=0.928), and blood pressure (P=0.526) between the three groups of crescents. Edema was the most prevalent symptom in all three groups, followed by oliguria and fever. Hemoptysis was significantly higher in patients with crescents ≥ 50% compared to the other two groups (P=0.011). Nephrotic syndrome and unexpected renal failure were significantly higher in patients with crescents ≤ 25% (P<0.001 and P=0.037, respectively). RPGN was significantly higher in patients with crescents ≥ 50% (P<0.001). A significant difference was observed in the incidence of nephritic syndrome among all three groups (P=0.037) (Table 1).

**Table 1:** Baseline demographic and clinicopathological characteristics of the patients Data are presented as numbers (%) unless otherwise specified. RPGN, rapidly progressive glomerulonephritis; SBP, systolic blood pressure; SD, standard deviation

Parameter	Crescent			P value
	≤25% (n=67)	26%-49% (n=35)	≥50% (n=43)	
Age (year), mean (SD)	31.91 (9.44)	33.77 (12.11)	34.27 (14.15)	0.540
Gender (female)	38 (56.72)	21 (60)	24 (55.82)	0.928
SBP (mmHg), mean (SD)	135.13 (16.15)	136.41 (16.18)	138.55 (13.57)	0.526
Presenting symptoms				
Edema	58 (86.56)	33 (94.28)	40 (90.69)	0.075
Oliguria	36 (53.73)	19 (54.28)	27 (62.79)	0.620
Fever	19 (28.35)	10 (28.57)	14 (32.55)	0.88
Rash	11 (16.41)	7 (19.99)	7 (16.27)	0.886
Arthralgia	19 (28.35)	8 (22.85)	10 (23.25)	0.766
Photosensitivity	13 (19.39)	10 (28.57)	10 (23.25)	0.580
Hemoptysis	1 (1.41)	0 (0)	5 (11.62)	0.011
Hematuria	9 (13.43)	8 (22.85)	19 (44.18)	0.001
Indication of biopsy				
Nephrotic syndrome	21 (31.34)	9 (25.71)	1 (2.32)	<0.001
RPGN	16 (23.88)	17 (48.57)	26 (60.46)	<0.001
Nephritic syndrome	12 (17.91)	3 (8.57)	11 (25.58)	0.004
Unexplained renal failure	8 (11.94)	4 (11.42)	2 (4.65)	0.037

**Figure 1:**
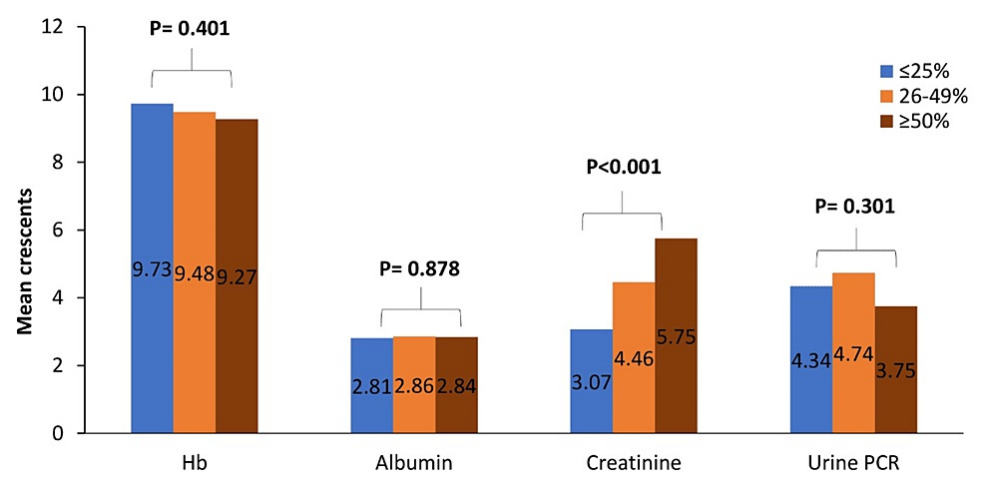
Laboratory findings of the study population Data are presented as percentages. Hb, hemoglobin; PCR, protein/creatinine ratio

The mean hemoglobin and mean albumin were comparable between all three groups. There was a significant difference observed in mean creatinine (P=0.001) and mean urine protein creatinine ratio (P=0.031) (Figure 1).

The levels of complement component 3 (C3), complement component 4 (C4), and antinuclear antibody (ANA) were comparable among all three groups. However, myeloperoxidase antineutrophil cytoplasmic antibody (MPO-ANCA), proteinase 3 ANCA (PR3-ANCA), and anti-glomerular basement membrane (anti-GBM) were significantly higher in the ≥50% crescent group (P<0.001, P<0.001, and P=0.004, respectively) (Table 2).

**Table 2:** Laboratory findings Data are presented as numbers (%). ANA, antinuclear antibody; ANCA, antineutrophil cytoplasmic antibody; GBM, glomerular basement membrane; MPO-ANCA, myeloperoxidase ANCA; PR3-ANCA, proteinase 3 ANCA; RBC, red blood cell

Parameter	Crescent			P value
	≤25% (n=67)	26%-49% (n=35)	≥50% (n=43)	
RBC casts in urine	12 (17.91)	3 (8.57)	11 (25.58)	0.004
C3 low	33 (49.25)	17 (48.57)	17 (39.53)	0.583
C4 low	17 (25.37)	8 (22.85)	8 (18.60)	0.715
ANA	19 (28.35)	10 (28.57)	10 (23.25)	0.817
ANCA				
PR3-ANCA	0 (0)	0 (0)	3 (6.57)	<0.001
MPO-ANCA	1 (1.49)	0 (0)	7 (16.27)	<0.001
Anti-GBM	0 (0)	0 (0)	2 (4.65)	0.004

On biopsy findings, there was no significant difference observed in mean glomerulosclerosis and mean interstitial fibrosis tubular atrophy (IFTA) among the three groups (P=0.117 and P=0.713, respectively). However, a significant difference was observed in mean crescents (P<0.001). In diagnosis, ANCA-associated vasculitis was significantly higher in the ≥50% crescent group compared to the other two groups (P<0.001). A significantly higher proportion of patients in the ≥50% crescent group required dialysis and plasmapheresis compared to the other two groups (P<0.001 and P=0.004, respectively) (Table 3).

**Table 3:** Primary diseases of the patients having GN with crescent Data are presented as numbers (%) unless otherwise specified. ANCA, antineutrophil cytoplasmic antibodies; GBM, glomerular basement membrane; GN, glomerulonephritis; IgA, immunoglobulin; IFTA, interstitial fibrosis tubular atrophy; SD, standard deviation

Parameter	Crescent			P value
	≤25% (n=67)	26%-49% (n=35)	≥50% (n=43)	
Biopsy finding (%), mean (SD)				
Glomerulosclerosis	23.40 (25.18)	21.92 (20.62)	15.06 (11.78)	0.117
IFTA	20.40 (13.26)	22 (12.54)	22.19 (10.92)	0.713
Crescents	15.58 (6.15)	34.57 (6.53)	72.03 (14.02)	<0.001
Crescent age				
Cellular	31 (46.26)	18 (51.42)	16 (34.88)	<0.001
Fibrocellular	11 (16.41)	18 (51.42)	12 (27.90)	
Fibrous	13 (19.40)	6 (17.14)	7 (16.27)	
Diagnosis				
IGA nephropathy	23 (34.32)	13 (37.14)	6 (13.95)	<0.001
Lupus nephritis	17 (25.37)	10 (28.57)	9 (20.93)	
ANCA-associated vasculitis	1 (1.49)	0 (0)	8 (18.60)	
Anti-GBM disease	0 (0)	0 (0)	2 (4.65)	
Dialysis	9 (13.43)	8 (22.85)	19 (44.18)	<0.001
Plasmapheresis	1 (1.49)	0 (0)	6 (13.95)	0.004

**Figure 2:**
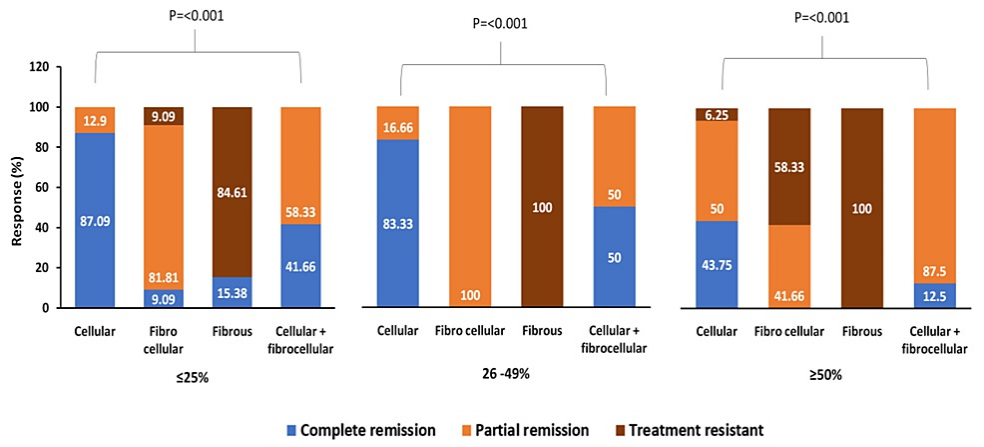
Response outcomes according to crescent age

Complete remission was observed in patients with ≤25% crescent (52.2%) and patients with 26%-49% crescent (48.6%), while partial remission was observed in patients with ≥50% crescent (46.5%). In the ≥50% crescent group, treatment resistance was significantly higher in fibrous crescent age compared to fibrocellular and cellular crescent age (100% versus 58.33% versus 6.25%; P<0.001). Complete remission was decreased in cellular crescent age with increasing the proportion of crescents (≤25% (87.09%) versus 26%-49% (83.33%) versus ≥50% (43.75%)) (Figure 2).

In the ≥25% group, normal renal function and urine analysis were observed in patients with cellular (87.09%) and a combination of cellular and fibrocellular (41.66%) crescent age. In the ≥50% crescent group, death was significantly higher in patients with fibrous crescent age (57.14%) (Table 4).

**Table 4:** Comparison of renal function and urine analysis according to crescent age Data are presented as numbers (%). CKD, chronic kidney disease; ESRD, end-stage renal disease

Crescents	Crescent age	Response					P value
		Normal renal function and urinalysis	Sustained proteinuria	CKD stage 3-4	ESRD	Death	
≥25%	Cellular (n=31)	27 (87.09)	2 (6.45)	2 (6.45)	0 (0)	0 (0)	<0.001
	Fibrocellular (n=11)	1 (9.09)	6 (54.54)	4 (36.36)	0 (0)	0 (0)	
	Fibrous (n=13)	2 (15.38)	0 (0)	7 (53.84)	3 (23.07)	1 (7.69)	
	Cellular + fibrocellular (n=12)	5 (41.66)	2 (16.16)	5 (41.66)	0 (0)	0 (0)	
26%- 49%	Cellular (n=18)	15 (83.33)	0 (0)	3 (16.66)	0 (0)	0 (0)	<0.001
	Fibrocellular (n=7)	0 (0)	2 (28.57)	5 (71.42)	0 (0)	0 (0)	
	Fibrous (n=6)	0 (0)	0 (0)	1 (16.66)	4 (66.66)	1 (16.66)	
	Cellular + fibrocellular (n=4)	2 (50)	0 (0)	2 (50)	0 (0)	0 (0)	
≥50%	Cellular (n=16)	6 (37.50)	5 (31.25)	4 (25.00)	1 (6.50)	0 (0)	<0.001
	Fibrocellular (n=12)	0 (0)	2 (16.66)	4 (33.33)	5 (41.66)	1 (8.33)	
	Fibrous (n=7)	0 (0)	0 (0)	0 (0)	3 (42.85)	4 (57.14)	
	Cellular + fibrocellular (n=8)	1 (12.5)	0 (0)	7 (87.5)	0 (0)	0 (0)	

## Discussion

GN with crescent is a severe manifestation of GN and represents the primary histopathological finding in cases of RPGN [15]. In the present retrospective study, GN with crescent accounted for a total of 145 patients over a period of five years, which is higher than the previous retrospective study observed in 49 patients within the same timeframe [2]. The predominance of females was observed in the current study, in alignment with the previous study conducted by Chen et al., which also reported a predominance of females (60.6%) in their study [17].

In the present study, the mean hemoglobin and albumin were comparable between all three groups. In contrast, the study done by Du et al. reported that the mean hemoglobin and albumin were significantly lower in patients with crescents ≥ 50% (P=0.001 and P<0.001, respectively) [18]. In the present study, the mean creatinine was significantly increased in patients with crescents ≥ 50% (P<0.001). A similar trend was observed in another study by Du et al., where the mean creatinine increased with an increasing proportion of crescent [18]. In the present study, C3 low and C4 low were comparable among all three groups. A previous study conducted by Wu et al. showed that the C3 and C4 were higher in anti-GBM GN with crescent [2]. In the current study, anti-GBM was observed in patients with crescents ≥ 50%. This result is similar to the study conducted by Yeter et al., which reported anti-GBM in five patients with crescents > 50% [3]. In the current study, the cellular crescent was significantly higher in all three groups compared to the fibrocellular and fibrous crescent (P<0.001). A study conducted by Parry et al. reported that fibrocellular crescents were the most common type of crescent seen in all three groups of GN with crescent [15].

Serum antineutrophil cytoplasmic antibody (ANCA) serves as a fundamental factor in identifying ANCA-associated vasculitis, a condition often characterized by type III GN with crescent (pauci-immune GN with crescent). It is worth noting that serum ANCA has also been observed in a limited number of patients with type I (anti-GBM) and II GN with crescent (granular immune complex deposition). Notably, research has shown that patients who tested negative for ANCA tended to have more favorable renal outcomes [2]. On the contrary, another study has indicated that individuals lacking ANCA exhibited elevated levels of proteinuria and more severe damage to the glomeruli and experienced worse kidney outcomes [17]. In the present study, ANCA-associated vasculitis was significantly higher in patients in the ≥50% crescent group compared to the other two groups (P<0.001). A study conducted by Parry et al. showed that ANCA positivity was seen in 87.9% of cases of type III crescentic GN [15].

A significantly higher proportion of patients with crescents ≥ 50% required dialysis and plasmapheresis compared to other groups (P<0.001 and P=0.004, respectively). A study conducted by Chaudhury et al. reported that 14 patients required dialysis at presentation [19]. Rampelli et al. showed that all pauciGN patients had renal limited disease; 5/8 showed positivity for p-ANCA. Plasmapheresis was given to 7/8 patients with pauciGN and one patient with anti-GBM disease [20].

In the current study, complete remission was observed in 18.6% of patients, and partial remission was observed in 46.5% of patients with ≥50% crescents. This is in contrast to a previous study conducted by Rampelli et al., who reported that in patients with >50% crescent, complete remission was seen in only 5.4% of patients, and no patients exhibited partial remission [20].

Nagaraju et al. reported that 34.5% of patients experienced complete or partial recovery. After six months, 31% of the patients depended on dialysis, and the mortality was 27.6% [21]. In the present study, the response and renal outcomes varied significantly with the crescent percentage and crescent age. Patients with ≥50% of crescents and fibrous crescents had poor response and renal outcomes.

Limitation

The present study has several limitations. First, the number of enrolled subjects was relatively small, potentially affecting the statistical power of the analyses conducted. Second, the cross-sectional design of the study may have introduced data interference, as there was no control population included for comparison. Furthermore, the study did not account for the potential influence of medications on the etiology, clinical features, histomorphology, and outcomes of GN with crescent. Additionally, it did not address variations in treatment among these groups and their influence on long-term outcomes.

## Conclusions

Crescent percentage and crescent age were significantly associated with a higher risk of renal failure and resistance to treatment. Hence, early diagnosis and treatment were associated with a better prognosis in patients with GN with crescent.
